# Predictors and risks of body fat profiles in young New Zealand European, Māori and Pacific women: study protocol for the women’s EXPLORE study

**DOI:** 10.1186/s40064-015-0916-8

**Published:** 2015-03-14

**Authors:** Rozanne Kruger, Sarah P Shultz, Sarah A McNaughton, Aaron P Russell, Ridvan T Firestone, Lily George, Kathryn L Beck, Cathryn A Conlon, Pamela R von Hurst, Bernhard Breier, Shakeela N Jayasinghe, Wendy J O’Brien, Beatrix Jones, Welma Stonehouse

**Affiliations:** School of Food and Nutrition, Massey University, Auckland, New Zealand; School of Sport and Exercise, Massey University, Wellington, New Zealand; Centre for Physical Activity and Nutrition Research, School of Exercise and Nutrition Sciences, Deakin University, Melbourne, Australia; Centre for Public Health Research, Massey University, Wellington, New Zealand; Office of Assistant Vice Chancellor Māori, Pacific & New Migrants, Massey University, Auckland, New Zealand; Institute of Information and Mathematical Sciences, Massey University, Auckland, New Zealand; Commonwealth Scientific and Industrial Research Organisation, Food and Nutrition Flagship, Adelaide, Australia

**Keywords:** Body fat profile, Predictors, Overweight and obesity, Metabolic disease risk, MicroRNA, Dietary practices, Physical activity, Taste perception, Women

## Abstract

**Background:**

Body mass index (BMI) (kg/m^2^) is used internationally to assess body mass or adiposity. However, BMI does not discriminate body fat content or distribution and may vary among ethnicities. Many women with normal BMI are considered healthy, but may have an unidentified “hidden fat” profile associated with higher metabolic disease risk. If only BMI is used to indicate healthy body size, it may fail to predict underlying risks of diseases of lifestyle among population subgroups with normal BMI and different adiposity levels or distributions. Higher body fat levels are often attributed to excessive dietary intake and/or inadequate physical activity. These environmental influences regulate genes and proteins that alter energy expenditure/storage. Micro ribonucleic acid (miRNAs) can influence these genes and proteins, are sensitive to diet and exercise and may influence the varied metabolic responses observed between individuals. The study aims are to investigate associations between different body fat profiles and metabolic disease risk; dietary and physical activity patterns as predictors of body fat profiles; and whether these risk factors are associated with the expression of microRNAs related to energy expenditure or fat storage in young New Zealand women. Given the rising prevalence of obesity globally, this research will address a unique gap of knowledge in obesity research.

**Methods/Design:**

A cross-sectional design to investigate 675 NZ European, Māori, and Pacific women aged 16–45 years. Women are classified into three main body fat profiles (*n* = 225 per ethnicity; *n* = 75 per body fat profile): 1) normal BMI, normal body fat percentage (BF%); 2) normal BMI, high BF%; 3) high BMI, high BF%. Regional body composition, biomarkers of metabolic disease risk (i.e. fasting insulin, glucose, HbA1c, lipids), inflammation (i.e. IL-6, TNF-alpha, hs-CRP), associations between lifestyle factors (i.e. dietary intake, physical activity, taste perceptions) and microRNA expression will be investigated.

**Discussion:**

This research targets post-menarcheal, premenopausal women, potentially exhibiting lifestyle behaviours resulting in excess body fat affecting metabolic health. These behaviours may be characterised by specific patterns of microRNA expression that will be explored in terms of tailored solutions specific to body fat profile groups and ethnicities.

**Trial registration:**

ACTRN12613000714785

## Background

Obesity has been described as an excess accumulation and storage of fat in the body, typically due to an increase in size and/or number of fat cells. Excess adiposity is generally accepted as an important risk factor for a range of non-communicable diseases; increased fat in the abdominal area compared with fat around the hips poses greater cardiovascular risks and metabolic dysregulation (Huxley et al. 2010; Martinez et al. 2008; Cameron et al. 2009). Yet, there has been no agreement on defining obesity in terms of body fat percentage (BF%) (Oliveros et al. 2014). A wide variety of BF% cut-off points have been used, varying between 20 to 25% for men and 30 to 37% for women (Oliveros et al. 2014). Alternatively, body mass index (BMI) is used worldwide to assess underweight, normal weight, overweight and obesity in adults of both genders (World Health Organisation 2011; Huxley et al. 2010; Gallagher et al. 1996; World Health Organisation 2000). However, BMI does not take into account body fat content or the differential health risks associated with abdominal (central) versus hip (peripheral) fat (Dulloo et al. 2010). There are also concerns regarding the acceptability of BMI as a reference value in varying ethnicities, as overweight and obesity have been defined based on European population data (Deurenberg 2001; World Health Organisation 2011, World Health Organisation 2000). Pacific people, Māori and Asian communities traditionally have larger and smaller body frames, respectively (Swinburn et al. 2004; Huxley et al. 2010; Deurenberg 2001; Rush et al. 2009; Ministry of Health NZ 2009) and subcutaneous fat patterns vary among ethnicities (Deurenberg and Deurenberg-Yap 2003; Okorodudu et al. 2010). Therefore, BMI is unlikely to accurately predict related disease risk profiles among different population subgroups with normal BMI or those that may have different levels of adiposity with a similar BMI (Gallagher et al. 1996; Deurenberg 2001; Ministry of Health NZ 2009).

Adiposity is likely to be overestimated in people with high BMI that have higher lean body mass (e.g. athletes), whilst underestimation is likely in those with lower BMI and less lean body mass (Oliveros et al. 2014; Romero-Corral et al. 2010; Okorodudu et al. 2010). The concept of metabolically obese normal weight individuals has been previously described (Karelis et al. 2004; De Lorenzo et al. 2006), and is associated with increased metabolic dysregulation. This has been referred to as “Normal Weight Obesity” (De Lorenzo et al. 2006) or as will be used in this study, a hidden body fat profile. However, not all individuals with the “hidden fat” profile may be at risk of metabolic disease, and it is unclear from previous studies whether metabolic dysregulation can be explained by high total fat mass, high BF% or high visceral fat (Oliveros et al. 2014).

During the transition from adolescence to adulthood (15–35 years), women experience larger weight gain than men (Jasik and Lustig 2008). Younger women often practice risky eating (e.g. dieting, fast foods) or lifestyle (e.g. physical inactivity) behaviours and work and environmental pressures may lead to altered food habits resulting in higher BF% that may negatively affect their metabolic profiles (Haslam and James 2005; Keskitalo et al. 2008). Furthermore, different taste sensitivities have been shown to influence dietary habits and metabolic health. A higher preference for sweet taste is associated with increased sugary food consumption (Drewnowski et al. 2012), whilst those who are hypersensitive to fatty acids consume less total fat (Stewart et al. 2010). Specific taste perception profiles may therefore lead to increased adiposity and ultimately reduced metabolic and cardiovascular health (Mendoza et al. 2007; Duffey and Popkin 2008). Gene-environment interactions may also increase the susceptibility of overweight individuals to develop hyperlipidemia, hypertension or diabetes (Martinez et al. 2008; Arkadianos et al. 2007). Excess body fat accumulation is mostly the result of a polygenic syndrome interacting with both dietary and physical activity components of lifestyle (Martinez et al. 2008). The signals delivered by food intake and physical activity regulate genes and proteins that alter energy expenditure/storage (Buttriss 2006). Gene/protein responses to food intake and physical activity vary between individuals; however, the cause is unknown (Martinez et al. 2008). MiRNAs are recently discovered molecules that act as “switches” to “turn on” or “turn off” genes and proteins. They are sensitive to diet and exercise (Güller and Russell 2010) and may influence the varied metabolic response seen between individuals (Davidsen et al. 2011). Recently, specific miRNAs have been suggested as biomarkers for metabolic disease (Heneghan et al. 2011). This unique gene-diet-physical activity relationship might impact on the development of hidden or apparent body fat, with different consequences across ethnic groups and thus requires further investigation (Swinburn et al. 2004; Arkadianos et al. 2007; Deurenberg and Deurenberg-Yap 2003; Di Renzo et al. 2007).

This study will be conducted as a cross-sectional comparative designed study. The primary aim of this study is to explore the metabolic risks and predictive factors associated with the hidden and apparent body fat profiles in 16 to 45 year old (post-menarcheal and premenopausal) NZ European, Māori and Pacific women. The primary outcomes are:

- Investigating the association between body composition profiles and markers of metabolic disease risk, including glucose, lipid and inflammatory marker profiles;
- Investigating dietary and physical activity patterns as predictive factors associated with body composition profiles;
- Investigating miRNA expression related to energy expenditure/storage as a predictive factor associated with body composition profiles.

The secondary outcomes are:

- Investigating associations/interactions between dietary and physical activity patterns and miRNA expression and how this may modulate the odds of having a specific body composition profile;
- Investigating taste perceptions as a predictive factor associated with the different body composition profiles;
- Investigating eating behaviour and habits as predictive factors associated with the different body composition profiles;
- Investigating nutrient intake as a predictive factor associated with the different body composition profiles.

We hypothesise that the “hidden fat” profile is associated with increased metabolic disease risk in NZ European, Māori, and Pacific women aged 16 to 45 years. We further hypothesise that for all women, dietary and physical activity patterns are predictors of a particular body composition profile by modulating miRNAs associated with energy expenditure/storage.

## Methods/Design

### Study design

The Women’s EXPLORE (“EXamining Predictors Linking Obesity Related Elements”) is a cross-sectional study targeting post-menarcheal, premenopausal NZ women to examine predictors of body composition profiles. Three body composition profile groups will be explored, namely:

“Normal Fat” group – normal BMI (<25 kg/m^2^), normal BF% (≥22%,<30%);

“Hidden Fat” profile group – normal BMI (<25 kg/m^2^), high BF% (≥30%);

“Apparent Fat” profile group – high BMI (≥25 kg/m^2^), high BF% (≥30%) (Oliveros et al. 2014; Okorodudu et al. 2010; NHLBI Obesity Education Initiative Expert Panel on the Identification Evaluation and Treatment of Overweight and Obesity in Adults 1998).

### Participants and sample size

Study participants are adult NZ women from three ethnic groups (NZ European, Māori, and Pacific Island). A total sample size of 225 women per ethnic group, consisting of 75 per profile group, will provide 80% power at significance levels of p < 0.05 to detect a medium effect size *f* of 0.25 (G*Power 3.1.2) for comparing the “hidden fat” profile with the other two body composition profiles (“normal fat” and “apparent fat”) regarding metabolic disease risk markers, dietary and physical activity patterns, and miRNA expression levels.

The medium effect size is relevant to all variables, and encompasses a variety of scenarios, as we wish to be able to explore how metabolic profile changes with body composition. For example, if the three groups have equally spaced means (μ - Δ, μ, μ + Δ), the difference in means will be detected with 80% power when Δ = 0.31 x σ, where σ is the within group standard deviation. For cholesterol, where preliminary data suggests σ = 0.98 mmol/L, we have 80% power to detect the difference when Δ = 0.30 mmol/L. Alternately, if two groups have the same mean μ and the third has mean μ + Δ, 80% power is achieved when Δ > 0.53 x σ, or 0.52 mmol/L in the case of cholesterol. A Δ = 0.30 – 0.52 mmol/L is estimated to be associated with a 9 – 15% lower relative risk of coronary heart disease (CHD)-related mortality (Gould et al. 2007). For HDL-C where preliminary data suggests σ = 0.38 mmol/L we have 80% power to detect a difference when Δ = 0.12 – 0.20 mmol/L. Every 0.1 mmol/L increase in HDL-C has been suggested to reduce CHD risk by between 8 – 15% (Gordon et al. 1989; Turner et al. 1998). For TG where preliminary data suggests σ = 0.45 mmol/L we have 80% power to detect a difference when Δ = 0.14 – 0.24 mmol/L. Studies in women showed a 1 mmol/L increase in TG was associated with 37% increase in risk of CVD (after adjustment for HDL-C and other risk factors)(Austin et al. 1998); Δ of 0.14 – 0.24 is thus estimated to be associated with 5.2 – 9% difference in CVD risk.

The power for simple (one variable) logistic regression for the risk of having a “hidden fat” profile among people of normal BMI is equivalent to the power of the independent sample t-test (Vaeth and Skovlund 2004) for comparing the predictor variable mean between the hidden fat and normal fat profiles. With a sample size of 75 per group and equal variance within groups, 80% power is achieved for differences of 0.46 σ. For instance, preliminary data on total energy expenditure assessed using the Recent Physical Activity Questionnaire (RPAQ) estimates the standard deviation at 5.36 METs-h/day; if the true difference means between the body composition groups is 2.47 METs-h/day, the logistic regression coefficient will be significantly non-zero 80% of the time. Clinically, the difference of 2.47 METs-h/day equates to ~684 kJ/day (164 kcal/day)(Besson et al. 2010), which is considered within the target range of recommended energy expended each day in physical activity and/or exercise (Pescatello and American College of Sports Medicine 2014). We will consider logistic regression predictors from the dietary and activity pattern data, and separately for the miRNA measurements.

**Figure 1:**
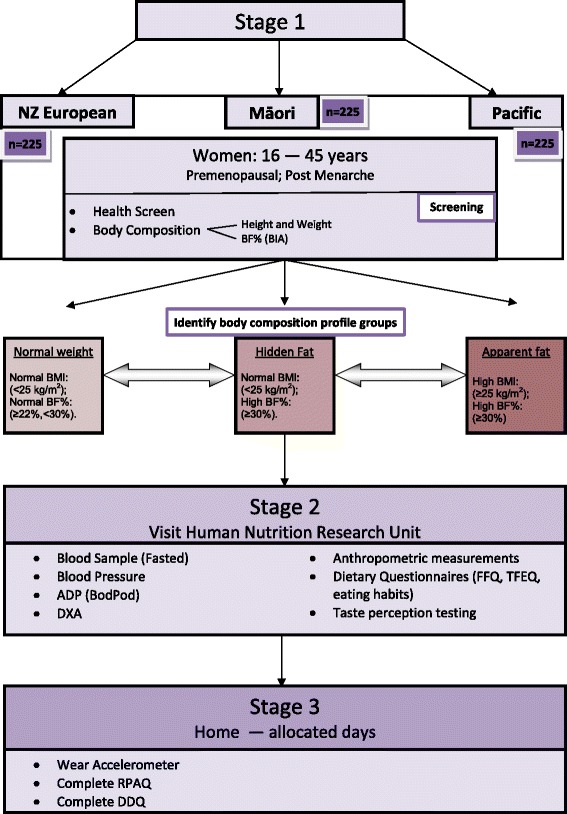
**Study design and procedures.**

Based on our pilot study that showed a prevalence of 21% of NZ European women having a “hidden fat” profile, (Kruger et al. 2010b) a sample of ~1140 women will need to be screened (380 per ethnicity) to find ~75 women per profile group; or to explore new profiles. The study design and study procedures are illustrated in Figure 1.

Inclusion criteria for women are:

- age (16 to 45 years),
- being post-menarcheal or pre-menopausal (as defined by a continuous regular menstrual cycle for the past one complete year),
- ethnicity (being of NZ European, Māori, or Pacific ethnicity as defined by self-identification and having at least one parent from the same ethnicity).

Exclusion criteria for women are:

- pregnancy and lactation,
- presence of any diagnosed chronic illness particularly affecting metabolic health (e.g. T2DM),
- presence of dairy allergy as the taste solution is dairy based.

### Setting and recruitment

Women are recruited in Auckland, NZ using media articles and advertising (e.g. in newspapers, magazines, on websites and radio interviews). Posters and flyers are used in a variety of venues including local crèches, primary schools, secondary schools, local businesses and events (e.g. gym’s, libraries, and sport events). The study is also advertised using social media (e.g. Facebook, twitter) and via emailing lists (e.g. Massey University staff, student, and previous research participant databases). Potential participants are directed to a study website for further information and to register their interest in the study. For the Māori and Pacific cohorts, recruitment strategies were adapted to be culturally appropriate. A more personal approach with face-to-face contact by community liaisons, were used to recruit women. Screening (see Stage 1 under procedures) was conducted within the respective communities and special assistance was provided for participants to travel to Massey University for further data collection (Stage 2).

### Procedures

The study follows a three-staged approach that involves screening (Stage 1), on-site assessments (Stage 2), and at-home assessments (Stage 3) (Figure 1).

#### Screening (stage 1)

Women who register interest are provided with an information sheet and asked to complete a consent form and the screening questionnaire. If all the inclusion and exclusion criteria assessed using the screening questionnaire is met, BMI (calculated using height and weight) and BF% (assessed using bioelectrical impedance (BIA)) are used for preliminary categorisation into the body composition profile groups (described above). The body composition screening is done in person and either at the Human Nutrition Research Unit, Albany campus, Massey University, or off-site.

#### Assessments (stages 2 and 3)

Participants recruited in stage 1 are invited to the research unit within 14 days of the start of their last menstrual period (the follicular phase) for testing to avoid the confounding effect of menstrual cycle hormones on taste perception, energy intake and energy expenditure (Davidsen et al. 2007).

Stage 2 of testing involves measurements of anthropometry, body composition, metabolic health, dietary intake, taste perception, and eating behaviour, which are described in detail below.

Stage 3 of assessment is completed at home over a seven day period. Participants wear accelerometers and keep a physical activity diary to assess physical and sedentary activity objectively in real life. At the end of the seven days, participants complete the RPAQ and the dietary diversity questionnaire (DDQ) (see Figure 1).

### Measures

The chosen measures and/or methods used for the various assessments in stages 1, 2 and 3 are listed in Table 1.

**Table 1:** **Measures and methods**

**Domain**	**Measures / methods**	**Reference**	**Equipment**	**Concept captured**
Body composition: anthropometry.	Anthropometric measurements (height, weight, circumferences) using ISAK protocol and standards.	(NHLBI Obesity Education Initiative Expert Panel on the Identification Evaluation and Treatment of Overweight and Obesity in Adults 1998)	Stadiometer, Lufkin tape.	Body composition.
				- Profile in terms of BMI (weight, height)
				- Risk in terms of circumferences and ratios (waist, hip, height).
Body composition profile – fat and lean mass.	-	(Ling et al. 2011)	Bioelectrical Impedance (BIA) (InBody230, Biospace Co. Ltd, Seoul).	Body composition - (fat and lean mass).
				- total
	-	(Noreen and Lemon 2006; Wingfield et al. 2014)	Air displacement Plethysmography (BodPod) (2007A, Life Measurement Inc, Concord, Ca., using software V4.2+ as supplied by the manufacturer).	Body composition - (fat and lean mass).
				- total
	-	(Boneva-Asiova and Boyanov 2008)	Dual XRay Absorptiometry (DXA) (Hologic QDR Discovery A, Hologic Inc, Bedford, MA. with APEX V. 3.2 software.	Body composition - (fat and lean mass).
				- total
				- regional
Metabolic health – biomarkers.	Analysis will be conducted by fully accredited laboratory with IANZ to the ISO 15189.	-	Blood sampling to capture plasma glucose, total cholesterol, triacylglyceride, HDL-cholesterol, LDL-cholesterol, insulin, serum hs-CRP, Il6, TNF-alpha, HbA1C, Leptin, Ghrelin.	Biomarkers related to metabolic health (lipid profile, glucose control, inflammation, hormonal control).
Metabolic health – blood pressure.	-	(Ogedegbe and Pickering 2010)	Blood pressure measurement Riester Ri-Chamion N digital blood pressure monitor, using one of two arm cuff sizes (22-32 cm or 32-48 cm).	Blood pressure related to metabolic health.
Metabolic health – gene expression	-	(Russell et al. 2012; Zacharewicz et al. 2014 )	miRNA - use specific primer and probes sets as per the manufacturer’s instructions (Applied Biosystems, Carlsbad, USA) using an MX3000p thermal cycler system. miRNA species will be measured using published techniques.	MiRNA related to energy expenditure.
Diet Quality	Food Frequency Questionnaire (FFQ)	(Ministry of Health NZ 1997; McNaughton 2011; Mishra et al. 2010)	Analysis using Foodworks7 2010 (Xyris Software (Australia) Pty Ltd, Queensland, Australia).	Dietary adequacy
				- energy intake
				- nutrient intake
				Patterns of food and nutrient intake.
Diet Quality	Eating Habits Questionnaire	Developed in this study	-	Dietary habits
				- eating habits
				- meal distribution
				- food choices.
Dietary Variety	Dietary Diversity Questionnaire (DDQ)	Developed in this study; mostly based on foods in the FFQ	-	Dietary diversity
				Food variety.
Dietary Behaviour	Three Factor Eating Questionnaire (TFEQ)	(Stunkard and Messick 1985)	-	Dietary behaviour
				- Restraint,
				- Disinhibition,
				- Hunger.
Physical Activity patterns	-	(Pescatello and American College of Sports Medicine 2014)	WGT3X Actigraph	Objective real life physical activity
				Physical activity expenditure
				- sedentary activities
				- intensity of activity.
	Physical Activity diary	-	-	Self-reported accelerometer non-wear time
				Self-reported intentional exercise
				– time
				- duration
				- type
				- intensity.
Physical Activity behaviour	Recent Physical Activity Questionnaire (RPAQ)	(Besson et al. 2010)	-	Self-reported physical activities
				- sedentary activities
				- time
				- intensity.
Taste perception, intensity, and hedonic preference	-	(Lim et al. 2008; Bartoshuk et al. 2004)	Rate intensity and hedonic preference of sweet and fat taste on a gLMS.	Sweet and fat taste sensitivity and preference.

#### Body composition assessment

Anthropometric measurements include weight, height, waist and hip circumferences using the International Society for the Advancement of Kinanthropometry (ISAK) protocol (Marfell-Jones et al. 2006). Waist-to-hip ratio, waist-to-height ratio and BMI will be derived from these measures and will be used to assess body composition in terms of obesity along with associated disease risk (NHLBI Obesity Education Initiative Expert Panel on the Identification Evaluation and Treatment of Overweight and Obesity in Adults 1998). Fat and lean mass are assessed using BIA (Boneva-Asiova and Boyanov 2008; Ling et al. 2011) for screening and preliminary categorisation into profile groups, and air displacement plethysmography (ADP) (using thoracic gas volume method) for final categorisation (Wingfield et al. 2014; Noreen and Lemon 2006), as well as DXA measurements for regional body composition (Boneva-Asiova and Boyanov 2008).

#### Metabolic health assessment

After an overnight fast (no food or beverages, excluding water for 12 hours prior to phlebotomy), blood samples are obtained by registered phlebotomists between 7.00 and 10.00 am and prior to sensory testing. Serum and plasma samples (ethylene diamine tetraacetic acid (EDTA) and heparin) are collected and processed in accordance with pathology laboratory protocols. All samples are frozen in separate aliquots in Eppendorf tubes and stored at −80°C until analysis. Analysis will be carried out by a fully accredited (with IANZ to the ISO 15189) laboratory or by qualified laboratory technicians upon completion of data collection. Biomarkers that will be analysed include plasma levels of glucose, total cholesterol, triglycerides, HDL-C, LDL-C, insulin, leptin, serum hs-CRP, Il-6 and TNF-alpha using commercially available kits or using routine enzymatic assays as published by our team (van Langenberg et al. 2014; Smith et al. 2012). The analyses will be performed using either a Biotek Synergy 2 Plate Reader (Millennium Science, Balwyn, Vic) or a Bioplex 200 plate reader (BioRad Hercules CA) depending on the required assay.

Selected miRNA species to assess metabolic disease risk and to explore energy expenditure and storage will be measured as published by our group (Russell et al. 2012; Güller and Russell 2010; Russell et al. 2013; Zacharewicz et al. 2014). For miRNA analysis, extracted RNA will be reverse transcribed using target specific primers followed by qPCR using target specific probes as published routinely by our group. All qPCR analysis will be performed using the Stratagene MX3000p thermocycler (Stratagene, La Jolla, CA); miRNA results will be normalized to RNA input and log transformed if not normally distributed.

Resting blood pressure measurements are taken whilst sitting, following a 10 minute resting period after taste perception assessments. For blood pressure measurements, an arm cuff is attached to the arm that has not been used for the venepuncture and three measurements are taken consecutively in one minute intervals (Ogedegbe and Pickering 2010).

#### Dietary intake data

A 220-item self-administered semi-quantitative food frequency questionnaire (FFQ) is used. The FFQ was adapted from the validated FFQ used in the Adult National Nutrition survey in NZ (Ministry of Health NZ 1997). Changes include an expanded food list to include currently consumed/available foods, additional questions relating specifically to fast food and snack food intakes, changes to the order of questions to improve continuity between questions. The food intake data will be processed using the Foodworks7 (Xyris Software (Australia) Pty Ltd, Queensland, Australia) dietary analysis database utilising FOODfiles 2010 (developed by the NZ Institute for Plant & Food Research and the NZ Ministry of Health) as the reference food composition table for NZ. Dietary quality and variety of the whole diet will be assessed, including nutritional adequacy and usual intake patterns and adherence to NZ dietary guidelines (Wirt and Collins 2009; Kant and Graubard 2005; Ibiebele et al. 2009). The frequency and patterns of intake of various nutrient-rich/poor dietary components and dietary factors that may affect fat deposition/obesity (e.g. sugar/fat-rich foods, fast foods, etc.) and related habits will be investigated. Foods from the FFQ will be grouped accordingly and factor analysis will be used to determine dietary pattern scores (McNaughton 2011; Mishra et al. 2010). Dietary variety will be assessed using dietary diversity scores and food variety scores to explore the variety of nutritious and non-nutritious foods (Ruel 2003; Murphy et al. 2006). Eating habits, meal patterns and food choices are explored using a self-developed and validated (during the current study) questionnaire, providing descriptive data of the women’s dietary practices.

Dietary behaviour is assessed using the validated TFEQ (Stunkard and Messick 1985; Bond et al. 2001) to measure three eating behaviour traits. The three factors are cognitive dietary restraint (Restraint), disinhibition of control (Disinhibition) and susceptibility to hunger (Hunger) by calculating scores for the dimensions and their sub-categories.

#### Sensory testing

Sensitivity and preferences of sweet and fat taste are assessed. Participants rate the intensity and hedonic preference of five sucrose and dairy samples on a general labelled magnitude scale (gLMS) (Bartoshuk et al. 2004; Lim et al. 2008).

#### Physical activity

A w-GT3X triaxial accelerometer (Actigraph, Pensacola, FL) secured to a waist belt, is worn for seven days, with a minimum of 10 h per day, to measure all components of physical activity and energy expenditure. Participants maintain their regular physical activity, whilst keeping a diary of times when the device is not worn. At least three full week and two weekend days is required for analysis. Physical activity counts will be calculated from raw data as the square root of the sum squared of activity counts, and will be categorized using metabolic equivalents (METs), as defined by the American College of Sports Medicine guidelines (Pescatello and American College of Sports Medicine 2014). Activities will be classified as light or sedentary (1.1-2.9 METs), moderate (3.0-6.0 METs), or vigorous (>6.0 METs). A physical activity diary is kept to record periods of non-wear and of intentional exercise. A self-reported RPAQ (Besson et al. 2010) is completed at the end of seven days with reference to the previous four weeks, allowing, together with the diary, a specific interpretation of the accelerometry counts.

### Data handling and statistical analysis

Statistical analysis will be performed using IBM SPSS statistics (IBM Corporation, New York, USA). Descriptive statistics will be used to describe the baseline population using mean (standard deviation), median (25, 75 percentile) or frequencies summary statistics. Normality of distribution will be evaluated using the Kolmogorov-Smirnov test and examining normality plots. Non-normally distributed variables will be transformed into approximately normal distributions by logarithmic transformations and again tested for normality. Primary statistical analyses will involve ANOVA tests with post-hoc analysis and Bonferoni adjustments comparing body composition profile groups regarding metabolic disease risk markers and dietary and physical activity patterns and miRNA levels; multiple logistic regression analysis to determine odds ratios of having a “hidden body fat” profile based on dietary and physical activity patterns; and, separately, miRNA expression levels. Principal components analysis will be performed on the miRNA data using the PCP directive in GenStat to identify linear combinations of the miRNAs that account for most of the variation between individuals (Zacharewicz et al. 2014); and Pearson correlations to determine correlation coefficients between dietary and physical activity patterns and miRNA expression profiles. A p-value of <0.05 will be considered significant.

### Ethics

Ethical approval was obtained from the Massey University Human Ethics Committee: (Southern A), Reference No.13/13.

## Discussion

An ethnic-specific focus aimed at weight maintenance has been recommended from previous NZ research (Metcalf et al. 2000). Overweight or obesity is a continual process and the effects of inappropriate lifestyle behaviour may often be missed when strictly assessing BMI as an indicator of over-nutrition. BMI provides only a crude measure of body fatness as it does not distinguish between weight associated with muscle or with fat. However, it appears to remain a useful estimate of the proportion of the population with increased risk of health conditions associated with obesity (World Health Organisation 2000), but may not be able to predict disease risk in those with normal BMI and varying adiposity.

Previous research has shown that a significant proportion of individuals with normal weight and subsequent normal BMI values had excessive body fat or “hidden” fat (Kruger et al. 2010a; De Lorenzo et al. 2006; Romero-Corral et al. 2010; Kruger et al. 2010b). This “hidden fat” profile may be linked with early inflammation and may be a key factor in the emerging epidemic of obesity and related disease risk (De Lorenzo et al. 2006; De Lorenzo et al. 2007). Pilot study data from our laboratory (Kruger et al. 2010a; Kruger et al. 2010b) revealed that in a free-living population (N = 116) of 18 to 44 year old NZ European women, 21.4% had a “hidden fat” profile and subsequent increased metabolic disease risk (identified through elevated fasting plasma leptin and insulin concentrations) and higher levels of sedentary lifestyle parameters.

Although a few studies have explored normal weight obesity (Marques-Vidal et al. 2008; Di Renzo et al. 2010; Romero-Corral et al. 2010; Karelis et al. 2004), not many studies have been specifically conducted to explore metabolic disease risk associated with high body fat (hidden or apparent), in premenopausal women. The typical central fat deposition following menopause and/or ageing may be absent in premenopausal women, despite the fact that women steadily gain weight from menarche to adulthood. It is therefore unclear where the hidden fat is situated in these women, especially if they are slender. It may be that excess fat in a lean person is hidden in the abdomen or elsewhere where it could pose health risks and cause metabolic dysregulation (Di Renzo et al. 2006; Huxley et al. 2010; Martinez et al. 2008; Cameron et al. 2009).

### Implications

The presence of normal weight obesity varies between 2 to 28% in women (Oliveros et al. 2014). The origins are unclear and may be due to environmental as well as genetic factors (Oliveros et al. 2014; Karelis et al. 2004). Common recommendations for appropriate dietary or lifestyle behaviours may be ineffective due to individual genetic variation. Dietary or physical activity behaviours may influence molecular mechanisms controlling metabolic activity and gene expression. MiRNAs can regulate the gene and protein networks that control substrate utilization and storage. Differences in behaviour that impact on miRNA expression need to be identified in different body composition profiles. Identifying these differences may assist in developing preventive recommendations (Arkadianos et al. 2007), and need to be specific to the locality and culture of the target population (Swinburn et al. 2004).

Because we do not know if and where slender women of different ethnicities with a normal BMI store hidden fat, it is important to identify this and its possible impact on women’s health. It is equally important to consider these issues in women with apparent fat and normal BF% to be able to make comparisons and explore tailored solutions. Our study will address a unique gap in health research knowledge by investigating multi-ethnic populations in NZ, identifying important physiological and behavioural predictors of metabolic disease risk. This research will assist in providing an important piece of the metabolic health versus body composition puzzle to fall into place and generate new pathways for treatment or early intervention.

## Abbreviations

ADP: Air displacement plethysmography
BF%: Body fat percentage
BIA: Bioelectrical impedance analysis
BMI: Body mass index
CVD: Cardiovascular disease
DDQ: Dietary diversity questionnaire
EDTA: Ethylene diamine tetra acetic acid
FFQ: Food frequency questionnaire
gLMS: General labelled magnitude scale
ISAK: International society for the advancement of kinanthropometry
miRNA: micro ribonucleic acid
METs: Metabolic equivalents
NZ: New Zealand
RPAQ: Recent Physical Activity Questionnaire
T2DM: Type two diabetes mellitus
TFEQ: Three factor eating questionnaire
